# Copper Does Not Induce Tenogenic Differentiation but Promotes Migration and Increases Lysyl Oxidase Activity in Adipose-Derived Mesenchymal Stromal Cells

**DOI:** 10.1155/2020/9123281

**Published:** 2020-02-20

**Authors:** Marta Milewska, Anna Burdzińska, Katarzyna Zielniok, Katarzyna Siennicka, Sławomir Struzik, Piotr Zielenkiewicz, Leszek Pączek

**Affiliations:** ^1^Department of Immunology, Transplantology and Internal Diseases, Medical University of Warsaw, 02-006 Warsaw, Poland; ^2^Department of Systems Biology, Institute of Experimental Plant Biology and Biotechnology, Faculty of Biology, University of Warsaw, 02-096 Warsaw, Poland; ^3^Institute of Biochemistry and Biophysics, Polish Academy of Sciences, 02-106 Warsaw, Poland; ^4^Department of Regenerative Medicine, Maria Sklodowska-Curie Institute-Oncology Center, 02-781 Warsaw, Poland; ^5^Department of Orthopedics and Traumatology, Medical University of Warsaw, 02-005 Warsaw, Poland

## Abstract

**Background:**

Copper belongs to the essential trace metals that play a key role in the course of cellular processes maintaining the whole body's homeostasis. As there is a growing interest in transplanting mesenchymal stromal cells (MSCs) into the site of injury to improve the regeneration of damaged tendons, the purpose of the study was to verify whether copper supplementation may have a positive effect on the properties of human adipose tissue-derived MSCs (hASCs) which potentially can contribute to improvement of tendon healing.

**Results:**

Cellular respiration of hASCs decreased with increasing cupric sulfate concentrations after 5 days of incubation. The treatment with CuSO_4_ did not positively affect the expression of genes associated with tenogenesis (*COL1α1*, *COL3α1*, *MKX*, and *SCX*). However, the level of COL1*α*1 protein, whose transcript was decreased in comparison to a control, was elevated after a 5-day exposition to 25 *μ*M CuSO_4_. The content of the MKX and SCX protein in hASCs exposed to cupric sulfate was reduced compared to that of untreated control cells, and the level of the COL3*α*1 protein, whose transcript was decreased in comparison to a control, was elevated after a 5-day exposition to 25 *μ*M CuSO_4_. The content of the MKX and SCX protein in hASCs exposed to cupric sulfate was reduced compared to that of untreated control cells, and the level of the COL3

**Conclusion:**

Copper sulfate supplementation can have a beneficial effect on tendon regeneration not by inducing tenogenic differentiation, but by improving the recruitment of MSCs to the site of injury, where they can secrete growth factors, cytokines and chemokines, and prevent the effects of oxidative stress at the site of inflammation, as well as improve the stabilization of collagen fibers, thereby accelerating the process of tendon healing.

## 1. Introduction

Tendinopathies, or tendon injuries, are a common medical problem mostly associated with their excessive use and aging. Unfortunately, despite treatment and a long period of rehabilitation, damaged tendons rarely reach full mechanical efficiency and strength [1]. This is due to the reduced cross-linking of collagen fibers and higher proportion of collagen type III (which has smaller diameter) to collagen type I in regenerated tissue in comparison with the normal tendon [2]. Since currently available therapies are not sufficient, new methods for effective treatment of tendon disorders are constantly being searched and old methods are being developed. There is a growing interest in transplanting mesenchymal stromal cells (MSCs) into the site of injury to improve the regeneration of damaged tendons. MSCs are characterized by their ability to differentiate into multiple cell types such as adipocytes, osteocytes, and chondrocytes. It is also possible to induce the tenogenic pathway in this population [3]. Besides their differentiation potential, MSCs can contribute to tendon healing through the secretion of many soluble factors such as growth factors (TGF*β* and HGF) and interleukins (IL-6, IL-8, and IL-10) [4] that act at the site of injury as chemoattractants for the cells involved in the tissue regeneration [5, 6]. MSCs can be isolated from various tissues, i.e., the bone marrow, adipose tissue, skin, and heart, and they possess the ability to regulate immune response which is useful in the case of inflammation occurring locally after injury of the tendon [7].

Copper (Cu) belongs to the trace metals that are essential for body homeostasis maintenance and play key roles in many basic cell functions regulating their metabolism [8]. It is known that copper is involved in growth and developmental processes, angiogenesis, iron metabolism, cellular respiration, and protection against oxidative stress [9, 10]. Some physiological functions of copper are associated with its role as a cofactor of cellular enzymes such as Cu/Zn superoxide dismutase (SOD1). The importance of copper homeostasis is demonstrated by numerous diseases associated with the impairment of its metabolism, i.e., neurological disorders, cardiomyopathy, tumorigenesis, Wilson's disease, and MEDNIK syndrome [11–14]. There is evidence proving that copper supplementation alters biological processes of MSCs. Rodriguez et al. [15] reported that it restrains proliferation of human BM-MSCs and stimulates their differentiation into osteocytes and adipocytes, whereas Li et al. [16] demonstrated that copper inhibits osteogenic differentiation of rat BM-MSCs. Therefore, it is possible that copper also affects the differentiation of MSCs towards tenocytes, especially considering that it is a cofactor of lysyl oxidase (LOX)—an enzyme involved in the cross-linking of collagen fibers [17].

The purpose of the study was to verify whether the supplementation of copper may have a positive effect on MSC's properties potentially contributing to improvement of tendon healing. Firstly, we have checked the impact of CuSO_4_ treatment on the ability of MSCs to differentiate into tenocytes. We have also investigated the effect of copper on the activity of intracellular superoxide dysmutase, responsible for protecting cells from the harmful effects of reactive oxygen species (ROS) [18] and lysyl oxidase, which is involved in the stabilization of elastin and collagen fibers in the extracellular matrix (ECM) [19]. Since the attempts are made to transplant MSCs into damaged tendons in order to improve their healing, we also aimed to verify if pretreatment of MSCs with copper compounds or simultaneous copper supplementation may have a beneficial effect on MSC motility/chemoattraction in order to facilitate their migration to the site of injury.

## 2. Materials and Methods

### 2.1. Human Adipose Tissue-Derived Stromal Cell (hASC) Isolation and Culture Methods

Human ASCs were kindly provided by Prof. Zygmunt Pojda whose group isolated the cells using the protocol described previously [3]. Adipose tissue was collected from healthy donors by liposuction. The samples were used in the studies after obtaining informed consent of the patients. The protocol that our group used for the identification of human adipose tissue stromal cells was previously described in detail [3]. Obtained cells were seeded on the cell culture dishes (8 × 10^4^ cells/cm^2^) in growth medium (GM): DMEM-low glucose (Biowest) supplemented with 10% fetal bovine serum (FBS; Biowest) and antibiotic-antimycotic solution (1.0% Penicillin-Streptomycin and 0.5% Amphotericin B; Invitrogen). Cells were cultivated under standard conditions (37°C, 5% CO_2_, and 95% humidity) until they reached the subconfluency. Afterwards, cells were trypsinized (0.25% Trypsin-EDTA solution; Invitrogen) and plated (5 × 10^3^ cells/cm^2^) for subsequent passage. The identification of MSCs was performed after the 4^th^ passage by flow cytometry analysis and multilineage differentiation. The remaining cells were frozen in liquid nitrogen, and MSCs at 5-6 passages were used for further experiments. Experiments were always conducted on cells from each donor separately. The cells from different donors were not pooled in this study to enable the detection of interindividual differences.

### 2.2. Human Tendon-Derived Cell Isolation and Culture

The tendon section was obtained during surgical procedure of a nonfunctional tendon section excision (correction of instability for, e.g., a tendon of the biceps muscle). The fragment was collected only from a patient who, regardless of this project, required a tendon intersection and its correction, so that the collection of the sample for examination did not affect the patient's state of health. The procedure was carried out in accordance with the approval of the Local Bioethics Committee and after obtaining the patient's informed consents. The tendon fragment after delivering to the cell culture lab was dissected into small fragments (about 1-2 mm^3^) and left on Ø 60 mm culture dish (BD Primaria™) in standard GM and cultured in standard culture conditions. The medium was partially replaced every 2-3 days without moving the tissue fragments. After 10 days, tissue fragments were collected, washed, and placed on a new dish for an additional 7-10 days. The cells which migrated from the tendon scrapes were harvested from the culture dish and reseeded on a new dish in a density of 3 × 10^3^ cells/cm^2^ for further growth. The cells were cultured up to passage 3, then harvested and cryopreserved.

### 2.3. Adipogenic Differentiation

To induce adipogenic differentiation, cells were cultured for 3 weeks in adipogenic medium consisting of DMEM-high glucose (Biowest) supplemented with 10% FBS, 1 *μ*M dexamethasone, 500 *μ*M 3-isobutyl-1-methylxanthine (IBMX), 10 *μ*g/ml insulin, 60 *μ*M indomethacin and 1% Penicillin-Streptomycin. The adipogenic medium was changed every third day. The differentiation was evaluated using Oil Red O staining which enables to visualize the lipid droplets. The cells were fixed with 4% paraformaldehyde for 10 min followed by 5 min incubation with 60% isopropanol and 15 min incubation with Oil Red O solution. After washing cells with distilled H_2_O, accumulated intracellular lipid droplets were visualized under light microscope.

### 2.4. Osteogenic Differentiation

To induce osteogenic differentiation, cells were cultured for 3 weeks in osteogenic medium consisting of DMEM-low glucose (Biowest) supplemented with 10% FBS, 100 nM dexamethasone, 10 mM *β*-glycerophosphate, 50 *μ*M L-ascorbic acid 2-phosphate, and 1% Penicillin-Streptomycin. The osteogenic medium was changed every third day. The differentiation was evaluated by visualization of calcium deposits using Alizarin Red staining. After 10 min of fixation with 4% paraformaldehyde and staining, cells were washed with distilled H_2_O and extracellular mineral deposits were visualized under light microscope.

### 2.5. Chondrogenic Differentiation

Chondrogenic differentiation was performed in a 15 ml tube. 7 × 10^5^ cells were suspended in chondrogenic medium consisting of DMEM-high glucose supplemented with 0.5% FBS, 100 nM dexamethasone, 1% insulin-transferrin-selenium solution (ITS), 100 *μ*M L-ascorbic acid 2-phosphate, 100 *μ*g/ml sodium pyruvate, 10 ng/ml TGF*β*2, and 1% Penicillin-Streptomycin and pelleted by centrifugation. After two days, formation of a cell spheroid structure at the bottom of the tube was observed. The chondrogenic medium was replaced every third day. After 3 weeks of culture, the cell spheroid structure was fixed with 4% paraformaldehyde for 30 min and immersed in paraffin for further histological analysis.

### 2.6. Flow Cytometry Analysis

After the 4^th^ passage, flow cytometry analyses (Becton Dickinson) of the cell-surface antigen profile were performed in order to identify MSCs. The presence of antigens CD44, CD73, CD90, and CD105 and the absence of CD11b, CD19, CD34, CD45, and HLA-DR were assessed using a Human MSC Analysis Kit (BD Stemflow) on BD FACSCanto II flow cytometer (Becton Dickinson).

### 2.7. Assessment of Cell Respiration

Cell viability was estimated using a 3-(4,5-dimethylthiazol-2-yl)-2,5-diphenyltetrazolium bromide (MTT) assay. The cells were cultured in GM with different concentrations (5, 10, 25, 50, 100, 500, and 1000 *μ*M) of CuSO_4_ (Sigma-Aldrich) for 5 days in a 96-well plate (4 × 10^3^ cells/well), and the MTT solution (final concentration 0.5 mg/ml) was added to each well 2 h before the end of the experiment. Next, the medium was aspirated and the chromogenic reaction product—formazan—was solubilized in dimethyl sulfoxide (DMSO, Sigma). The absorbance corresponding to the activity of mitochondrial dehydrogenase was measured on the BioTek PowerWave XS microplate reader at a wavelength of 570 nm.

### 2.8. Evaluation of the Cell Viability under Hydrogen Peroxide-Induced Oxidative Stress

The cells were seeded in a 96-well plate (4 × 10^3^ cells/well) and left to attach for 24 h. After 5 subsequent days of preincubation of hASCs in GM supplemented with CuSO_4_ (concentrations: 0 *μ*M, 50 *μ*M, and 100 *μ*M), cells have been subjected to oxidative stress induced by hydrogen peroxide (final concentration 1 mM in growth medium). After 24 hours, cell viability was assessed with MTT test as described above.

### 2.9. RNA Isolation and Quantitative Real-Time PCR

For gene expression analysis, human ASCs were seeded on Primaria™ cell culture dishes (Ø 60 mm) at a density of 5 × 10^3^ cells/cm^2^ in GM. Once they reached about 80% of confluence, they were exposed to GM with 25 *μ*M or 100 *μ*M CuSO_4_ for 5 days. The isolation of total RNA was performed using an RNeasy Mini Kit (Qiagen). RNA concentration and purity were assessed spectrophotometrically with a NanoDrop ND-1000 (NanoDrop Technologies, Inc.). The conversion of total RNA on cDNA was conducted using a PrimeScript RT reagent kit (Takara) according to the producer's instructions. Real-time PCR was performed in triplicates on an ABI Prism 7500 Sequence Detection System (Applied Biosystems) using Premix Ex Taq (Takara) master mix for real-time PCR and specific TaqMan primers and probes (Applied Biosystems): collagen type I alpha 1 (*COL1Α1*) Hs00164004_m1, collagen type III alpha 1 chain (*COL3A1*) Hs00943809_m1, scleraxis (*SCX*) Hs03054634_g1, and Mohawk homeobox (*MKX*) Hs00543190_m1. Tyrosine 3-monooxygenase/tryptophan 5-monooxygenase activation protein zeta (*YWHAZ*) Hs03044281_g1 was used as a housekeeping gene. Each sample was analyzed in duplicate. The relative gene expression was calculated by the 2^−*ΔΔ*Ct^ method. The reference sample was always the untreated cells from the same donor cultured in parallel. Cells from 3 donors were used in each of the two separate experiments performed.

### 2.10. Western Blot Analysis

For analysis of the cellular content of examined proteins, human ASCs were seeded on Primaria™ cell culture dishes (Ø 100 mm) in a density of 5 × 10^3^ cells/cm^2^ in GM. Once they reached about 80% of confluence, they were exposed to GM with 25 *μ*M or 100 *μ*M CuSO_4_ for 5 days. Untreated cells from the same donors were cultured in parallel as a control. In order to receive whole cell lysates, a RIPA buffer (Sigma-Aldrich) with protease and phosphatase inhibitor cocktails (Sigma-Aldrich) were used. Protein concentration in the lysates was assessed using a Bio-Rad protein assay dye reagent according to the manufacturer's instructions (Bio-Rad Laboratories Inc.). The same amount of proteins per sample (50 *μ*g) was applied to the gel and resolved by SDS-PAGE. Proteins were then transferred on the PVDF membrane. After subsequent blocking of the membranes with 5% nonfat dry milk in TBST buffer, membranes were incubated overnight with appropriate primary antibody solution: rabbit polyclonal anti-collagen I antibody (Abcam, ab34710, 1 : 500), rabbit polyclonal anti-collagen III antibody (Abcam, ab7778, 1 : 500), rabbit polyclonal anti-Mohawk antibody (LSBio, aa46-75, 1 : 1000), rabbit polyclonal anti-Scleraxis antibody (Thermo Fisher Scientific, PA5-23943, 1 : 1000), or rabbit polyclonal anti-actin antibody (Santa Cruz Biotechnology, sc-1616-R, 1 : 250). Next, the membranes were washed 3 times for 15 min with TBST and incubated with secondary antibodies (dilution 1 : 5000) conjugated with near-infrared (IR) fluorophores (IRDye 800CW or IRDye 680RD). The membranes were analyzed using ChemiDoc MP Imaging System (Bio-Rad Laboratories Inc.) and the integrated optical density (IOD) of each band was quantified using Image Lab software (Bio-Rad Laboratories Inc.). Cells from 3 donors were used in each experiment. Western blot analysis was performed in triplicate from three independently executed experiments.

### 2.11. Determination of the Relative Levels of Secretory Activity (Proteome Profiler)

The comparison of the cytokine secretion profiles between control hASCs, cupric sulfate-treated hASCs, and human tendon-derived cells (TCs) was performed using Proteome Profiler, a membrane-based antibody array that enables to simultaneously determine the relative levels of 102 human cytokines in a variety of biological materials. For this assay, cells were cultured with/without 50 *μ*M CuSO_4_ for 5 days. For the last 48 h of the experiment, the growth medium was replaced with the serum-free medium, i.e., DMEM-low glucose supplemented with 3.5% bovine serum albumin and antibiotic solution (1.0% Penicillin-Streptomycin) with/without cupric sulfate. In parallel, tendon-derived cells were cultured in the same conditions (without CuSO_4_). Supernatants were then collected and frozen in aliquots at -80°C. To perform the Proteome Profiler, ASC supernatants from two independent donors were pooled and further steps were performed according to the manufacturer's instruction.

### 2.12. Transwell Migration Assay

The effect of CuSO_4_ treatment on the directed migration of hASCs was studied using cell culture inserts with 8 *μ*m pore size (Thincert™, Greiner). This protocol is based on the method previously described by our group [20]. In the basal compartment (bottom of the 24-well plate), unlabeled ASCs from each donor (*n* = 3) were separately seeded in a concentration of 1.5 × 10^4^/well. Next day after seeding, cells were treated with CuSO_4_ in concentrations of 25, 50, or 100 *μ*M. Cells cultured in GM served as a control. The test was performed in duplicates. After 3 days, inserts were placed on each well. Cells from 2 different ASC populations were pooled and stained with red fluorochrome DilC18(5)-DS (1,1′-dioctadecyl-3,3,3′,3′,-tetramethylindodicarbocyanine-5,5′-disulfonic acid, DID), Ex = 650 nm and Em = 670 nm (AAT Bioquest). This pool constituted a migrating population in the experiment and was the same for all samples. These cells were seeded in the concentration of 1.5 × 10^4^ on each insert. Plates with inserts were incubated at 37°C and 5% CO_2_ for the next 48 h to allow cell migration from the inserts to the basal compartment. Afterwards, inserts were removed, cells from the wells were fixed with 4% paraformaldehyde (10 min, RT), and the cell nuclei were visualized with DAPI staining (20 ng/ml of DAPI solution for 4 minutes, RT). Additionally, migrating cells were trypsinized from the bottom side of inserts; they were allow to attach overnight and also fixed and stained with DAPI. The cells were visualized with imaging reader Cytation™ 1 (BioTek) and counted with Gen5 3.04 software. The number of migrating/stimulating cells was calculated from 6 different fields of view in each well. Migrating cells were identified based on red fluorescence (DID staining). The stimulating population assessment was based on the number of nuclei minus the number of DID-stained cells. The migrating cells constituted the sum of these that fell from the insert to the well bottom and these that were detached from the underside of an insert. Fields of view had the same locations in all wells and were chosen arbitrarily before analysis. The ratio of migrating cell numbers (with red fluorescence, [Supplementary-material supplementary-material-1]') to the stimulating cell numbers (unlabeled, [Supplementary-material supplementary-material-1]”) was calculated in accordance to the following equation:

1 $$\begin{array}{c} \text{Stimulation potential}=\frac{\left(\text{DIDf}+\text{DIDins}\right)}{\left(\text{DAPI}-\text{DIDf}\right)}, \end{array}$$

DIDf is the number of migrating cells (stained with DID) which fell from the insert, DIDins is the number of migrating cells (stained with DID) which were detached from the underside of an insert, and DAPI is the number of cell nuclei (stained with DAPI). Nuclei of stimulating cells and DIDf cells were counted.

At least 5000 migrating cells per each treatment were included into the analysis.

### 2.13. SOD1 Activity Assay

The activity of intracellular superoxide dismutase (SOD) was evaluated using a commercially available colorimetric test (Superoxide Dismutase Colorimetric Activity Kit; Invitrogen) with xanthine oxidase according to the manufacturer's protocol. Xanthine oxidase generates superoxide anions, which catalyze the formation of a purple product. This reaction is inhibited by superoxide dismutase. In brief, hASCs were seeded in a density of 5 × 10^3^ cells/cm^2^ in GM on Primaria™ cell culture dishes (Ø 100 mm) and were treated with CuSO_4_ when they reached about 80% confluence. After 5 days of hASC exposure to 25 *μ*M or 100 *μ*M cupric sulfate, the cells were lysed using RIPA buffer without detergents (50 mM Tris and 150 mM NaCl) with protease and phosphatase inhibitor cocktails (Sigma-Aldrich). The substrate solution was added to cell lysates and standards on the 96-well plate, and the absorbance values at 450 nm were measured in order to blank the plate. Next, the xanthine oxidase solution was added to the analyzed wells in a ratio of 1 : 2 to the substrate. After a 20-minute incubation at room temperature, the absorbance was measured on an Infinite 200 PRO microplate reader (Tecan) at 450 nm. The activity of SOD is inversely proportional to the absorbance values and it was referenced to the standard curve. In each sample, the protein concentration was measured using Bio-Rad protein assay dye reagent (Bio-Rad Laboratories Inc.) in order to normalize the SOD activity. The standards and samples were analyzed in triplicate.

### 2.14. LOX Activity Assay

Lysyl oxidase enzymatic activity was estimated using a commercially available fluorometric assay (Lysyl Oxidase Activity Assay Kit; Abcam) according to the manufacturer's instruction. Briefly, 5 × 10^3^ cells/cm^2^ were seeded on Primaria™ cell culture dishes (Ø 100 mm) and after reaching about 80% confluence they were cultured with 25 *μ*M or 100 *μ*M CuSO_4_ for 5 days. Cell lysates were received using RIPA buffer without detergents (50 mM Tris and 150 mM NaCl) with protease and phosphatase inhibitor cocktails (Sigma-Aldrich) and were applied on a 96-well plate in triplicates. The LOX Reaction Mix has been prepared and added to cell lysates in a 1 : 1 ratio. After 30 min incubation (37°C, protecting from light), fluorescence intensity was measured on an Infinite 200 PRO microplate reader (Tecan) at Ex/Em = 535/595 nm. In every sample, the activity of LOX was normalized to the protein concentration measured using a Bio-Rad protein assay dye reagent (Bio-Rad Laboratories Inc.). Cells from 3 donors were used in three independent experiments.

### 2.15. Statistical Analysis

All the statistical analyzes were conducted using STATISTICA software (StatSoft®). First, the data distribution within groups was analyzed using a Shapiro-Wilk test. The groups of related data with abnormal distribution were analyzed using Wilcoxon matched-pairs signed-rank test. Student's *t*-test was used to compare value groups with confirmed normal distribution. Significance was set at *p* < 0.05, and graphs are presented as mean ± standard error of the mean (SEM).

## 3. Results

**Figure 1:**
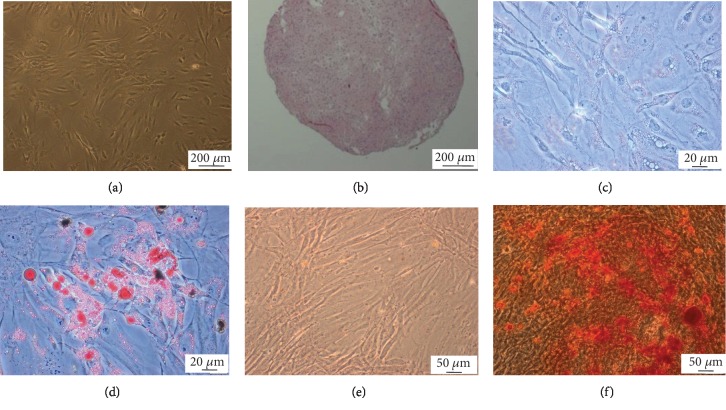
Human ASC morphology and differentiation potential. Light microscopy: (a) undifferentiated hASCs; (b) chondrogenic differentiation, HE staining of chondropellet; (c, d) adipogenic differentiation, Oil Red O staining (lipid droplets are red) of hASCs cultured in standard (c) and adipogenic (d) medium; (e, f) osteogenic differentiation, Alizarin Red staining (calcium deposits are red) of hASCs cultured in standard (e) and osteogenic (f) medium. Scale bars: 200 *μ*m (a, b), 20 *μ*m (c, d), and 50 *μ*m (e, f).

The adipose tissue-derived cells from three healthy donors were collected and seeded on plastic culture dishes. Cells adhered to the surface of the dish and formed colonies. The mean expression of CD44, CD73, CD90, and CD105 amounted to 99.6%, 97.8%, 83.9%, and 99.7%, respectively (*n* = 3 donors). The mean expression of hematopoietic markers (a cocktail of CD11b, CD19, CD34, CD45, and HLA-DR) amounted to 1.98%. Multilineage differentiation capacity of isolated hASCs has been confirmed by successful differentiation into adipocytes, osteocytes, and chondrocytes (Figure 1).

### 3.1. The Effect of Cupric Sulfate on hASC Respiration

**Figure 2:**
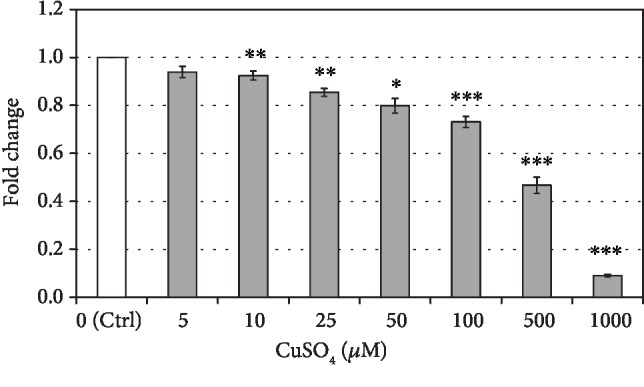
The effect of cupric sulfate on hASC viability measured in MTT test. Data presented as means ± SEM. Analyzed using tests for related groups in comparison to the control (Ctrl); ^∗^*p* < 0.05, ^∗∗^*p* < 0.01, and ^∗∗∗^*p* < 0.001; *n* = 6‐9.

Cellular respiration of hASCs decreased with increasing cupric sulfate concentrations after 5 days of incubation, and this decrease was statistically significant for the CuSO_4_ concentration of 10 *μ*M and all higher concentrations. The drop of cell viability for 10, 25, 50, 100, 500, and 1000 *μ*M of CuSO_4_ amounted to 7.6%, 14.6%, 20.2%, 26.9%, 53.3%, and 90.9%, respectively, in comparison to control, untreated cells (Figure 2).

### 3.2. The Effect of Cupric Sulfate on Tenogenic Differentiation of hASCs

**Figure 3:**
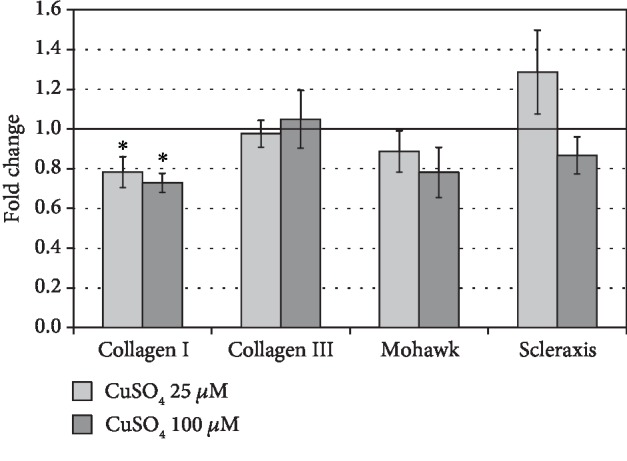
The effect of cupric sulfate on the *COL1α1*, *COL3α1*, *MKX*, and *SCX* expression assessed by qPCR analysis in hASCs. The expression of analyzed genes in control cells was appointed as 1. Data presented as means ± SEM. Analyzed using tests for related groups in comparison to the control; ^∗^*p* < 0.05; *n* = 6.

**Figure 4:**
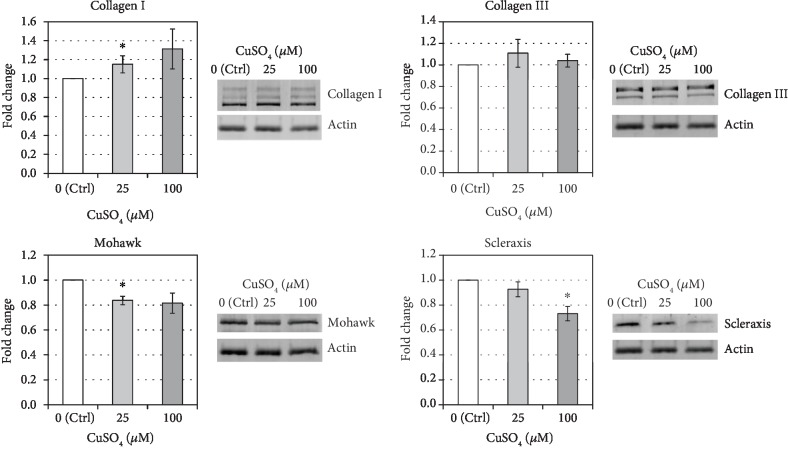
The effect of cupric sulfate on the COL1*α*1, COL3*α*1, MKX, and SCX protein level assessed by immunoblotting in hASCs. Data presented as means ± SEM. Analyzed using tests for related groups in comparison to the control (Ctrl); ^∗^*p* < 0.05; *n* = 11‐14.

After 5 days of exposition on cupric sulfate (25 *μ*M and 100 *μ*M), the expression of *COL1α1* in hASCs was reduced in comparison to untreated control cells (mean drop by 21.7% and 27.1%, respectively, both *p* = 0.02). Simultaneously, there were no significant differences in the expression of other genes associated with tenogenesis, i.e., *COL3α1*, *MKX*, and *SCX* (Figure 3). On the other hand, the protein level of COL1*α*1 was slightly elevated after the 5-day treatment of hASCs with cupric sulfate and this increase was statistically significant for 25 *μ*M CuSO_4_ (fold change = 1.15 vs. control, *p* = 0.03). The MKX and SCX protein content in adipose tissue-derived stromal cells exposed to cupric sulfate was decreased compared to that in untreated control cells (for MKX—mean drop by 16.3% in 25 *μ*M CuSO_4_-treated cells, and for SCX—mean drop by 26.8% in 100 *μ*M CuSO_4_-treated cells vs. the control group, respectively, both *p* < 0.05). However, the COL3*α*1 protein level remained unchanged (Figure 4).

### 3.3. Cupric Sulfate Changes the Profile of Cytokine Secretion by hASCs

**Figure 5:**
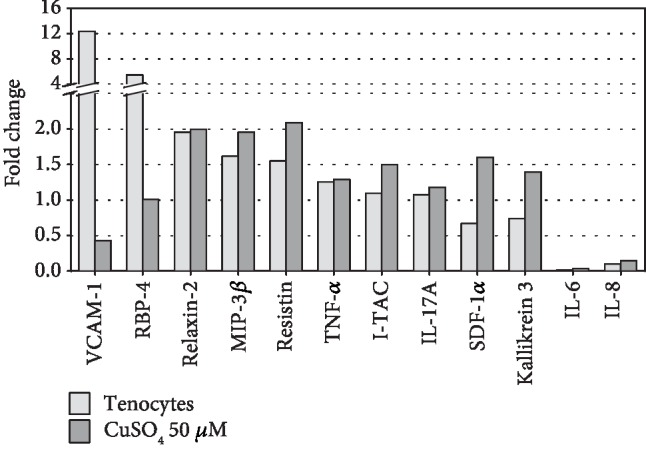
The comparison of cytokine secretion profile of human tendon-derived cells and cupric sulfate-treated human adipose tissue-derived stromal cells relative to control (untreated hASCs) assessed by the Proteome Profiler. The secretion of analyzed cytokines in control cells was appointed as 1. Data presented as means.

Human tendon-derived cells showed in general distinctly lower secretory activity compared to untreated and cupric sulfate-treated hASCs. The secretion of only 8 out of 102 analyzed cytokines was higher in tenocytes in comparison to untreated hASCs. They were VCAM-1 (fold change = 12.36), RBP-4 (fold change = 5.44), relaxin-2 (fold change = 1.95), MIP-3*β* (fold change = 1.62), resistin (fold change = 1.55), TNF-*α* (fold change = 1.26), I-TAC (fold change = 1.09), and IL-17A (fold change = 1.07). Interestingly, the secretion of all abovementioned cytokines, except for VCAM-1, was also elevated in hASCs exposed for 5 days to 50 *μ*M cupric sulfate compared to that of the control cells. Moreover, CuSO_4_ treatment increased the secretion of SDF-1*α* (fold change = 1.60 vs. control) and kallikrein 3 (fold change = 1.39 vs. control). On the other hand, both tenocytes and CuSO_4_-treated hASCs showed significantly reduced secretion of interleukins, IL-6 (fold change = 0.01 and 0.04 vs. control, respectively) and IL-8 (fold change = 0.10 and 0.14 vs. control, respectively) (Figure 5).

### 3.4. Cupric Sulfate Increases the Migratory Abilities of hASCs

**Figure 6:**
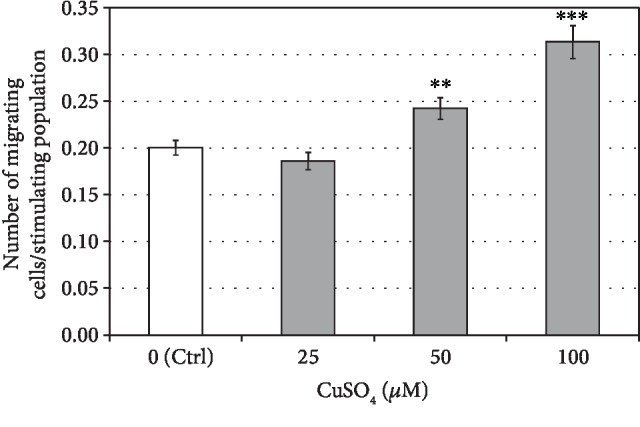
The effect of cupric sulfate on migratory abilities of human adipose stromal cells. Data presented as means ± SEM. Analyzed using a *t*-test for related groups in comparison to the control (Ctrl). ^∗∗^*p* < 0.01 and ^∗∗∗^*p* < 0.001. Cells from 3 donors used in analysis.

Transwell migration assay showed that exposition of human adipose tissue-derived stromal cells to the cupric sulfate stimulated their motility and it was positively correlated with the concentration of CuSO_4_. The number of migrating cells in relation to the stimulating population treated with cupric sulfate increased by 21% and 56% in comparison to control untreated cells at a CuSO_4_ concentration of 50 and 100 *μ*M, respectively (Figure 6). The low concentration (25 *μ*M) of cupric sulfate did not alter the migratory abilities of hASCs.

### 3.5. Cupric Sulfate Positively Regulates the Activity of Superoxide Dismutase SOD1 in hASCs

**Figure 7:**
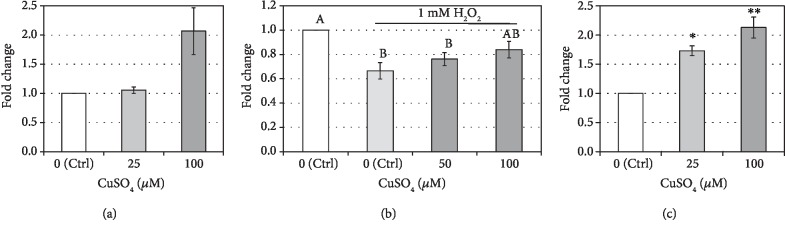
The effect of cupric sulfate on copper-dependent enzyme activity in hASCs. (a) The effect of cupric sulfate on superoxide dismutase SOD1 activity. Data presented as means ± SEM; *n* = 3. (b) The effect of cupric sulfate on hydrogen peroxide-induced oxidative stress damage measured in MTT test. Data presented as means ± SEM. Statistical significance is indicated by letters: there is no significant difference between groups with the same letter (*p* > 0.05). Analyzed using tests for related groups in comparison to the control (Ctrl) + 1 mM H_2_O_2_; *n* = 6‐9. (c) The effect of cupric sulfate on lysyl oxidase activity. Data presented as means ± SEM. Analyzed using tests for related groups in comparison to the control (Ctrl); ^∗^*p* < 0.05 and ^∗∗^*p* < 0.01; *n* = 8‐9.

Addition of cupric sulfate in high concentration (100 *μ*M) for 5 days of culture resulted in stimulation of intracellular superoxide dismutase (SOD1) activity (fold change = 2.07 vs. control) whereas CuSO_4_ in low concentration (25 *μ*M) did not alter the activity of the copper-containing enzyme (Figure 7(a)). To define whether increased SOD1 activity in hASCs treated with high concentration of CuSO_4_ is associated with increased resistance to oxidative stress, we determined cell viability after 24 h incubation with 1 mM H_2_O_2_. Hydrogen peroxide-induced oxidative stress significantly decreased cell respiration in untreated hASCs (*p* < 0.05). The drop of viability under oxidative stress in cells preincubated previously with 50 *μ*M cupric sulfate was slightly less profound but still significant in comparison to control cells cultured in GM without exposure to H_2_O_2_ (*p* < 0.05). The preincubation of cells with 100 *μ*M CuSO_4_ resulted in higher resistance to oxidative stress—the viability of these cells was not significantly lower than the viability of untreated cells (*p* > 0.05); however, it was also not significantly higher than the viability of cells without preincubation with cupric sulfate and exposed to H_2_O_2_ (Figure 7(b)).

### 3.6. Cupric Sulfate Stimulates the Activity of Lysyl Oxidase (LOX) in hASCs

Treatment of hASCs with cupric sulfate for 5 days of culture resulted in increased lysyl oxidase activity and was positively correlated with the concentration of CuSO_4_ (Figure 7(c)). The exposition of MSCs to low doses of cupric sulfate (25 *μ*M) resulted in increased LOX activity by 73% (*p* < 0.05) in comparison to control cells whereas after treatment with high concentrations of CuSO_4_ (100 *μ*M), the enzyme activity increased 2.13-fold (*p* < 0.01).

## 4. Discussion

Intensive research work on the properties of mesenchymal stromal cells has been underway for many years. Since the discovery of their ability to differentiate into many cell types, their immunomodulatory properties and secretion of various cytokines and growth factors, MSCs have begun to be perceived as a promising tool for therapeutic purposes, including tissue and organ regeneration. As mentioned, MSCs can be isolated from various tissues, and their most common sources are bone marrow and adipose tissue. In our studies, we used the adipose tissue-derived mesenchymal stromal cells, since obtaining this cell type is easier, less invasive, and more efficient than the isolation of MSCs from the bone marrow [21]. It is believed that besides their well-documented ability to differentiate into osteocytes, adipocytes, and chondrocytes, which is simultaneously one of the criteria that cells must meet to be considered as MSCs [22], these cells can also differentiate into other cell types, e.g., tenocytes. However, to date, the markers specific for tenocytes only have not been established. A characteristic feature of tenocytes is the production of a large amount of extracellular matrix (ECM), in particular, type I collagen and, in smaller amounts, type III collagen. However, these proteins are expressed in many types of cells. There are also several transcription factors that play important roles in the development of tendons, including scleraxis (Scx), which is involved in the differentiation and organization of ECM in tendons, [23] and Mohawk (Mkx), which is essential in the processes of tendon growth and collagen fiber maturation [24]. We have evaluated the effects after 5 days of treatment based on the literature data indicating that changes in gene and protein expression were detectable both at the 3^rd^ and the 7^th^ day of tenogenesis [3, 25].

In the presented study, we have examined the influence of copper supplementation on tenogenic differentiation of hASCs by determining the expression of transcription factors (SCX and MKX) and collagen types I and III at the level of transcript and protein products. The treatment with cupric sulfate did not positively affect the expression of molecules associated with tenogenesis (Figure 3). However, the level of type I collagen protein, whose transcript was decreased in comparison to the control, was elevated after the 5-day exposition to 25 *μ*M CuSO_4_ (Figure 4). Similarly, Héraud et al. have demonstrated that copper supplementation increased collagen synthesis in human articular chondrocytes [26]. We hypothesized that changes in collagen expression under influence of copper compounds might be associated with affected activity of lysyl oxidase. It is known that LOX stabilizes ECM structure initiating the process of cross-linking of collagen fibers [19]. Moreover, historical data indicate that there is a positive dose-dependent relationship between the dietary copper and lysyl oxidase activity in chick bone and tendon [27]. And indeed, our results clearly demonstrate that the activity of LOX in hASCs was significantly elevated under the influence of CuSO_4_ and was positively correlated with its concentration (Figure 7(c)). Interestingly, Atsawasuwan et al. demonstrated that LOX directly interacts with TGF*β*1 and suppresses its signaling through SMAD3 [28]. Furthermore, Havis et al. showed that TGF*β*/SMAD2/3 signaling was involved in differentiation of mouse mesodermal stem cells towards the tendon lineage ex vivo and in vitro [29]. These results taken together might be a clue in the explanation of a decrease in MKX and SCX production by hASCs under influence of CuSO_4_ observed in this study. It is possible that increased activity of LOX inhibits TGF*β* signaling (MSCs are known to secrete TGF*β* which act in an autologous and paracrine way). This inhibition could result in suppressed expression of tendon-associated transcription factors. However, this is only a hypothesis which would require further verification. On the other hand, increased LOX activity after influence of CuSO_4_ could facilitate the stabilization of collagen produced during the healing of an injured tendon. Even if transplanted ASCs would not differentiate into tenocytes, the secreted LOX can act on collagen synthetized by other cells. This might accelerate the process of tendon repair.

High hopes for the use of mesenchymal stromal cells for therapeutic purposes are associated not only with their ability to multilineage differentiation, but also with secretion of various cytokines acting as chemoattractants for other cells whose presence is necessary in tissue regeneration processes. Although there were no positive changes in expression of molecules directly associated with tenogenesis under CuSO_4_ treatment, the secretion profile of the hASCs after the 5-day exposure to 50 *μ*M cupric sulfate differed from untreated cells and was similar to the secretion profile of human tenocytes (Figure 5). Among the cytokines with increased secretion in adipose tissue-derived stromal cells exposed to CuSO_4_, there was a stromal cell-derived factor-1*α* (SDF-1*α* and CXCL12) which is known to promote migratory abilities of stromal cells [30, 31]. As the increased motility of MSCs is essential for their recruitment to the site of tendon injury, we checked whether cupric sulfate is able to positively influence the migration process of hASCs. Indeed, the transwell migration assay demonstrated that CuSO_4_ stimulates the motility of adipose tissue-derived MSCs (Figure 6). It is probable that this effect was due to higher secretion of CXCL12 as these results are in agreement with the increased secretion of this chemokine after treatment with CuSO_4_ (Proteome Profiler analysis, Figure 5). These results find confirmation in the latest findings of Chen et al. showing that copper supplementation enhances migration on bone marrow-derived mesenchymal stromal cells [32]. The authors demonstrated that the action of cupric sulfate is associated with the cytoskeleton remodeling induced by hypoxia-inducible factor 1*α*- (Hif1*α*-) dependent upregulation of the rho family GTPase 3 (Rnd3). Our results suggest additional explanation of this phenomenon—the increased CXCL12 secretion. It is possible that two mentioned mechanisms play a role in the promoted MSC's migration under influence of CuSO_4_. Our results in this area seem to be particularly interesting as CXCL12 is known to attract not only mesenchymal stromal cells, but also other stem and progenitor cells [33]. It was demonstrated that local increase in CXCL12 concentration promotes the healing of the injured tendon. Shen et al. [34] showed that CXCL12 incorporated into knitted silk-collagen sponge scaffold enhanced the efficiency of tendon regeneration by accelerating the recruitment of fibroblast-like cells and tendon extracellular matrix production in comparison to the scaffold without CXCL12.

Interestingly, another protein with increased secretion present in both CuSO_4_-treated ASCs and tenocytes compared to untreated hASCs was relaxin-2. Although relaxin is considered primarily as a pregnancy-related hormone, there is evidence indicating that it may influence peripheral tissues, for example, the tendon—reducing its stiffness by collagenase activation and decreasing tissue collagen content [35].

Cupric sulfate supplementations have also influenced the activity of another copper-dependent enzyme in human adipose tissue-derived stromal cells. The SOD activity assay showed that the addition of high level of CuSO_4_ to the culture medium increases the action of intracellular superoxide dysmutase (Figure 7(a))—the enzyme protecting cells from the harmful effects of ROS occurring in case of inflammation and tissue damage. This can be beneficial for tendon healing after the injury, as increased ROS levels may induce autophagy in transplanted cells which in turn can switch from the pathway leading to survival to the path of the cell death in adverse conditions [36]. The obtained result is consistent with the findings that both dietary and injected copper elevated SOD activity in rat erythrocytes [37] and that CuSO_4_ solution injection increased SOD activity in the rat liver [38]. It was also observed that the higher were the doses of cupric sulfate, the higher was the survival rate of hASCs under oxidative stress conditions, although these results did not reach the statistical significance in comparison to cells exposed to oxidative stress without preincubation with CuSO_4_ (Figure 7(b)). This could be caused by the fact that the oxidative stress was induced by hydrogen peroxide which is a product, not a substrate, of the superoxide dismutation, the reaction catalyzed by SOD [39]. Moreover, in the presence of copper ions, H_2_O_2_ can be converted to hydroxyl radical in Fenton reaction [40].

There are more and more studies concerning the use of MSC transplants in order to improve the healing of the injured tendon. Summarizing our results, we cannot state that copper sulfate treatment can be used as an additional factor positively affecting tenogenesis in human adipose tissue-derived stromal cells; however, certain concentrations of CuSO_4_ can stimulate migratory abilities of hASCs potentially increasing their effectiveness in reaching the site of injury, where they can contribute to the tendon healing through their immunomodulatory properties. Moreover, the exposure to CuSO_4_ changes the secretion profile of adipose tissue-derived MSCs and increases the activity of copper-dependent enzymes—lysyl oxidase involved in the stabilization of collagen fibers and superoxide dismutase preventing the effects of oxidative stress and inflammation in cells. Therefore, cupric sulfate supplementation may have beneficial effects on tendon regeneration not through the induction of tenogenic differentiation but through the improving the recruitment of human adipose-derived stromal cells to the site of injury where they can secrete numerous growth factors and cytokines acting as chemoattractants, thus accelerating the healing process.

## Data Availability

The datasets used and/or analysed during the current study available from the corresponding author on reasonable request.

## Supplementary Materials

Supplementary Materials Images from automatic imaging reader Cytation™ 1: A, A', and A” present the same field of view. Cells migrating from the upper compartment of the inserts are stained with DID (red color); cell nuclei are stained with DAPI (blue color). A' and A” demonstrate the method of analysis (A'—nuclei numbering, and A”—migrating cell numbering); scale bar: 1000 *μ*m.
