# Supplementary material for: Copper Does Not Induce Tenogenic Differentiation but Promotes Migration and Increases Lysyl Oxidase Activity in Adipose-Derived Mesenchymal Stromal Cells
Source: Stem Cells Int. 2020 Feb 20;2020:9123281. doi: 10.1155/2020/9123281 (PMC7053469; doi:10.1155/2020/9123281)
Supplement: Supplementary Materials — Images from automatic imaging reader Cytation™ 1: A, A', and A” present the same field of view. Cells migrating from the upper compartment of the inserts are stained with DID (red color); cell nuclei are stained with DAPI (blue color). A' and A” demonstrate the method of analysis (A'—nuclei numbering, and A”—migrating cell numbering); scale bar: 1000 μm. [file 9123281.f1.pdf]

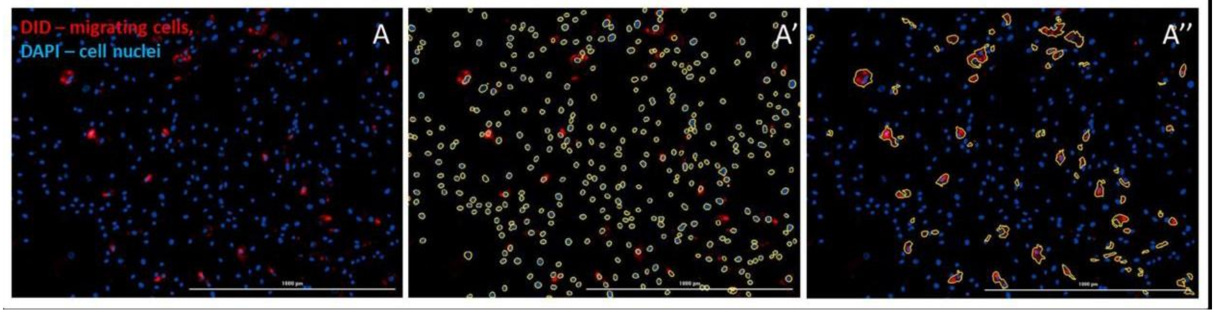

Supplementary material. S1. Images from automatic imaging reader Cytation™ 1. A, A’, A’’ present the same field of view. Cells migrating from the upper compartment of the inserts are stained with DID (red color), Cell nuclei are stained with DAPI (blue color). A’ and A’’ demonstrate the method of analysis (A’ – nuclei numbering, A’’ – migrating cells numbering), scale bar – 1000 µm.
